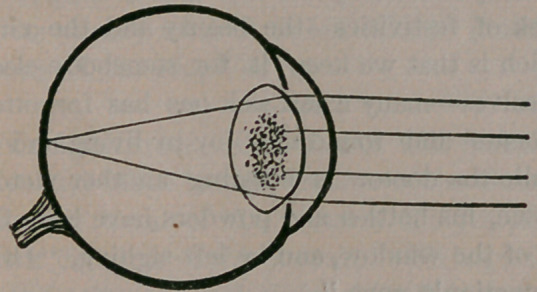# Glaucoma, Artificial Pupil

**Published:** 1876-01

**Authors:** 


### GLAUCOMA.

ARTIFICIAL PUPIL.

The name *glaucoma* is given to one of the most dangerous diseases of the eye with which the ophthalmic surgeon has to deal, when not speedily recognized, and the proper treatment applied. Should the patient fall into the hands of a physician unfamiliar with the symptoms and consequences of *glaucoma*, irremediable blindness would be the certain result; for, only in the *beginning* of the disease, can blindness be averted, by means of the operation which we are about to describe. It would therefore seem to be of the utmost importance that not only the family physician, but the laity as well, should be able to recognize the symptoms that invariably accompany threatened blindness of this character, to the end that an ophthalmic surgeon's advice and skill can be sought and procured before the disease is thoroughly established.

Formerly, hundreds of people became incurably blind from this insidious disease, in spite of the various forms of treatment that had been recommended by the most learned men in ophthalmic science. It was not until after the celebrated German oculist, Helmholtz, had invented the ophthalmoscope—in 1851—(the wonderful instrument by means of which we are permitted to examine the entire internal structure of the eye, and to easily recognize any abnormal appearance of humors, retina, or nerve,) that the still greater oculist, Von Grafe, discovered the true nature of glaucoma and revealed an operation for its cure.

Without entering into a lengthy explanation as to the manner in which blindness is caused by this disease, suffice it to say that it is the result of extreme tension of the eye-ball, by which the humors within the eye press upon the retina and nerve, producing paralysis of those parts, and consequent blindness. The manner in which this pressure is relieved, will appear when describing the operation. The symptoms ushering in the disease, are rapidly increasing far-sightedness, requiring the use of stronger and stronger glasses, to enable the patient to read, and periodical attacks of dimness of vision. In looking at a lamplight a halo or rainbow, appears about it. The eyes feel full, and the patient complains of a pressure about them, as though *something* in them was about to burst. Considerable pain is felt, at times, about the temples, over the brow, and in the eye-ball.—Later, the field of vision becomes contracted and distorted, the patient not being able to see over so large a space, and some times can only see part of an object; as, in looking at a man, will see his face but not his body, or will distinguish his nose and eyes, but cannot see the forehead and chin.—At first, these symptoms only come on now and then, perhaps weeks and even months intervening between the attacks. As the disease progresses, however, they become more frequent, when only days or hours will separate the attacks from each other.

Usually, no external changes in the appearance of the eye manifest themselves, so that the pains are apt to be considered rheumatic or neuralgic, and the aberration of vision will be thought but a temporary affair, to pass off with the pains and aches. From this reasoning, many fatal mistakes have occured, and we desire to urge our readers against committing such a terrible blunder.

A careful observer, in looking at an eye so afflicted, will notice the pupil to be slightly enlarged, and the *iris* sluggish in its movements, not responding readily to bright light. The pupil will not be clear, but in persons past forty or forty-five years—after which time of life it is most prevalent—will present a yellowish-green hue. If you press upon the eye-ball, over the closed lids, with your finger, it will seem hard and unyielding, and these are the only appearances that are manifested externally. Usually, no inflammation or redness of the ball is present, and to the casual observer, the eye would appear sound.

But, let the ophthalmic surgeon view the eye with his ophthalmoscope, and he will tell you of enlarged vessels in the retina, of blood effused under the retina or other membranes, from ruptured vessels; of changes in the appearance of the optic nerve, &c., &c., all of which would be Greek to you, who only desire to know—"can it be cured?" Happily, it can, and by means of the most delicate operation known to surgeons, and called by them *iridectomy*, or the formation of an "artificial pupil." The operation consists in puncturing the *cornea* with a thin spear-shaped knife, near its margin, making an opening sufficiently large through which to introduce a delicate forceps or hook, and, grasping the *iris* at its pupiliary margin, drawing it through the wound made in the cornea, and snipping it off with the scissors. The procedure will be better understood by introducing this cut:

The illustration shows the opening in the iris made in its side next the nose. It is usually made for *glaucoma*, at its upper margin, so that the slight deformity may be hidden under the lid.—The operation of snipping out a portion of the *iris* relieves the *intra-ocular* pressure, as oculists term it; that is, it prevents the too abundant supply of the aqueous humor—which is discharged at the time of the operation, and so relieves the internal pressure in the eye-ball,—giving relief to the sensitive membrane that suffered through this pressure. Immediately, that the operation is performed, all pain and other unpleasant symptoms about the eye subside, the rainbow, luminous spots, and floating stars all disappear, and the patient is almost immediately restored to vision. Cannot, therefore, an operation that will relieve pain, blindness, and other distressing symptoms, instantly, be considered a brilliant success? We think so, and believe that no patient will long delay in seeking this aid, who is suffering from the symptoms here detailed.

*Iridectomy*, or artificial pupil, is performed often quite as successfully for other affections of the eye, as when the pupil becomes obstructed with lymph, the result of *iritis* or other inflammation of the internal structures. In such cases, when atropia is instilled into the eye, the pupil will be found to dilate unevenly, presenting the forms and shapes seen below :

depending entirely upon the number and location of the little bands of lymph which hold the *iris* tied to the lens, and so preventing its expansion. Sometimes these deposits are so dense as to resemble *cataract*, and to almost wholly exclude the light from the eye. When this is the case, a portion of the *iris* is removed in the same manner as described for *glaucoma*, when the pupil will appear as here illustrated, so that the light can pass through the new pupil, above the natural one, where the obstruction exists. In this manner, vision is again speedily restored. Opacities of the cornea—spots or "films" on the clear part of the eye—are often so large and dense as to shut out light from the pupil. In such cases, the same operation is performed, provided there remains any portion of the cornea clear enough to allow the light to pass to the new pupil when it is made.

There is a form of *cataract*, very slow in reaching complete blindness, in which an operation for artificial pupil is sometimes made, by means of which, useful vision is retained for many years.—How this can be, will be best understood by introducing another cut :

It will be observed that the central portion of the crystaline lens has become opaque, so that the rays of light reaching the center of the eye, are arrested the moment they reach the lens, where the opacity exists, while those that fall upon the margin are refracted and reach the retina, as shown in our engraving. Now, to snip a piece of the iris out, opposite the *clear* margin of the lens, will permit more rays of light to reach the fundus of the eye and so restore good vision.

From what has now been written upon this operation of artificial pupil, it will be perceived what a valuable means for restoring sight it must be when employed by the skillful operator, and we hope that the subject has been made sufficiently plain and comprehensive, to induce the family physician to recommend patients presenting the symptoms here described, to the ophthalmic surgeon, early, in order that his skill may be used to avert the irremediable blindness that is sure to follow neglect or improper treatment.

## Figures and Tables

**Figure f1:**
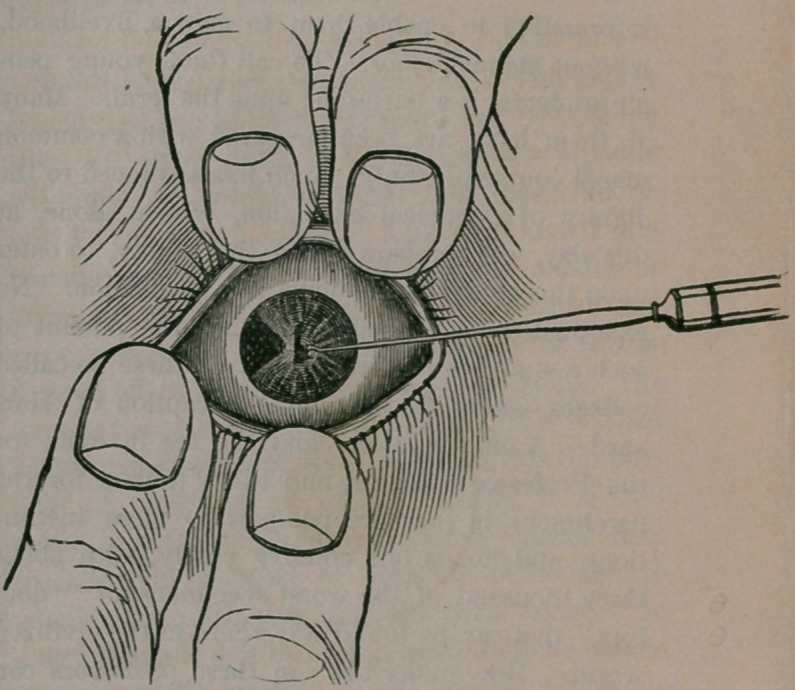


**Figure f2:**
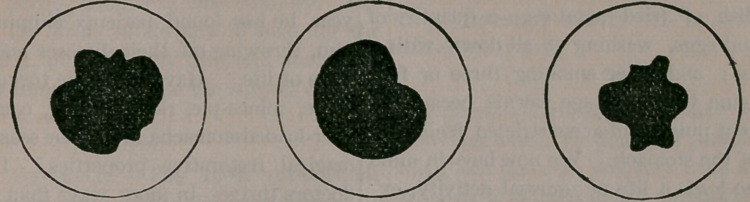


**Figure f3:**
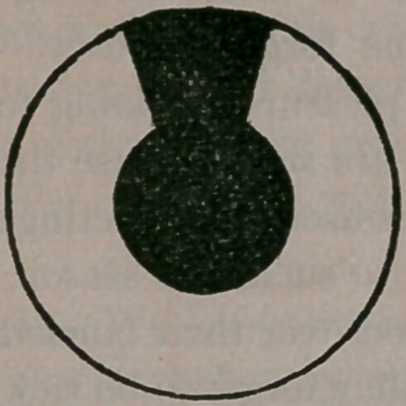


**Figure f4:**